# Stable Isotope Anatomy of Tropical Cyclone Ita, North-Eastern Australia, April 2014

**DOI:** 10.1371/journal.pone.0119728

**Published:** 2015-03-05

**Authors:** Niels C. Munksgaard, Costijn Zwart, Naoyuki Kurita, Adrian Bass, Jon Nott, Michael I. Bird

**Affiliations:** 1 Research Institute for the Environment and Livelihoods, Charles Darwin University, Darwin, Northern Territory, Australia; 2 Centre for Tropical Environmental and Sustainability Science, College of Science, Technology and Engineering, James Cook University, Cairns, Queensland, Australia; 3 Graduate School of Environmental Studies, Nagoya University, Nagoya, Japan

## Abstract

The isotope signatures registered in speleothems during tropical cyclones (TC) provides information about the frequency and intensity of past TCs but the precise relationship between isotopic composition and the meteorology of TCs remain uncertain. Here we present continuous δ^18^O and δ^2^H data in rainfall and water vapour, as well as in discrete rainfall samples, during the passage of TC Ita and relate the evolution in isotopic compositions to local and synoptic scale meteorological observations. High-resolution data revealed a close relationship between isotopic compositions and cyclonic features such as spiral rainbands, periods of stratiform rainfall and the arrival of subtropical and tropical air masses with changing oceanic and continental moisture sources. The isotopic compositions in discrete rainfall samples were remarkably constant along the ~450 km overland path of the cyclone when taking into account the direction and distance to the eye of the cyclone at each sampling time. Near simultaneous variations in δ^18^O and δ^2^H values in rainfall and vapour and a near-equilibrium rainfall-vapour isotope fractionation indicates strong isotopic exchange between rainfall and surface inflow of vapour during the approach of the cyclone. In contrast, after the passage of spiral rainbands close to the eye of the cyclone, different moisture sources for rainfall and vapour are reflected in diverging d-excess values. High-resolution isotope studies of modern TCs refine the interpretation of stable isotope signatures found in speleothems and other paleo archives and should aim to further investigate the influence of cyclone intensity and longevity on the isotopic composition of associated rainfall.

## Introduction

The use of isotopes to reconstruct long-term, high-resolution records of tropical cyclones (TCs) is a relatively recent advance within the developing field of palaeotempestology. Tropical cyclone rainwater, compared to monsoonal and thunderstorm rain, is typically depleted in ^18^O and ^2^H due to extensive isotopic fractionation of atmospheric moisture flowing towards the TC core. To date, this fingerprint has been used to develop annual records of TCs extending back over 1500 years in Australia [1,2] and at weekly intervals over a 26 year period in Belize [3] from cave speleothems. These records are registered following the percolation of ^18^O depleted rainwater through the cave roof, dissolving limestone which precipitates as generally seasonal layers of speleothem calcite between 100 and 200 μm thick. The same TC isotope fingerprint is also preserved in tree ring cellulose [4] and has been used to generate tree-ring records of past TC activity over the last 200 years in the south-eastern USA [5].

While existing speleothem and tree ring isotope proxy records compare well with historical records of TCs for these regions there still remains uncertainty around the precise relationship between the isotope signature registered by the proxy and meteorological parameters of the TC. These parameters include the TC intensity, longevity of the system, distance from the sampling location to the TC eye or track and distance inland from the coastal crossing location and associated progressive weakening of system intensity and persistence of the isotopic signature within the rainfall.

Following the pioneering work in the Gulf of Mexico [6–8] there have been relatively few studies examining the isotope values of TC (or ex-TC) rainfall over a substantial portion of the life of a TC system or along the TC track [9, 10]. Of particular relevance are the characteristics of the isotope values after the cyclonic system crosses the coast and begins to weaken, as sampling locations can be some distance inland from the coast. There is also little available data on the relationship between isotope values and various structural aspects of TCs such as spiral bands and zones in between and variations in relative humidity and rainfall rates.

The energy driving the circulation of TCs is provided by the evaporation of moisture from the sea surface and the subsequent release of latent heat upon the condensation of water vapour which also generates precipitation [6]. Moisture is conveyed along the surface towards the TCs low-pressure core and inside a radius of about 100 km from the core moisture inflow is typically 10-fold greater than the moisture flux from the surface within the central area itself [11]. As a consequence, the O and H isotope anatomy of TCs is influenced not only by the physical processes within the cyclone itself but also by the moisture sources and the precipitation histories of the air masses that become entrained in the circulation system [10,12,13].

We present here the results of an investigation into the O and H isotope characteristics of rainfall generated during TC Ita, which made landfall in northeast Queensland on April 11th, 2014. After landfall, TC Ita travelled over land parallel to the coast and re-entered the Coral Sea ~ 300 km south of its initial landfall location. Samples of rainfall were collected along the length of this track at a variety of time intervals over a two-day period. The most intense sampling was undertaken near Cairns where, for the first time, two Isotope Ratio Infrared Spectrometers (IRIS) were used to simultaneously obtain continuous real-time δ^18^O & δ^2^H values of both rainfall and water vapour during the approach and passage of a TC. The results allow us to draw conclusions about the characteristics of the isotope values along the cyclone track and over time after making landfall. Comparisons between isotope values and rainfall rates, relative humidity and moisture source areas were also possible. The results are important for not only understanding the isotope variability within a TC over time but also for testing specific conclusions made about a previously derived ~ 800 year long TC isotope record collected close to the track of this system [2].

## Observations

**Fig 1:**
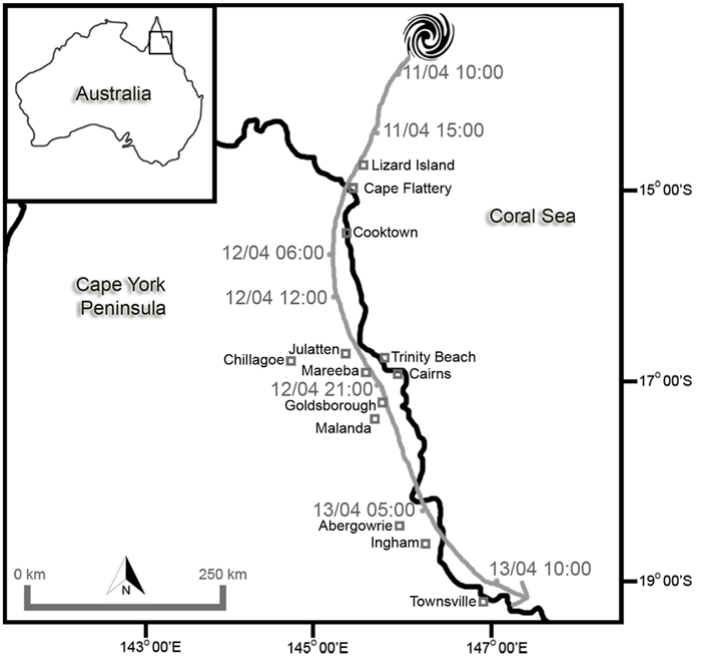
Sampling locations and track of TC Ita April 11 to 13, 2014 [14, 15].

Tropical Cyclone Ita developed from a tropical low on 1 April 2014 over the Solomon Islands and gradually moved westward. Banding features wrapped around the circulation and deep convection became persistent by 2 April. On 10 April, Ita intensified into a Category 5 system on the Australian Scale (central pressure ~ 930 hPa), but weakened to a Category 4 prior to landfall at Cape Flattery in North Queensland at 11 April 22:00 Australian Eastern Standard Time (AEST) (Fig. 1). Following landfall, Ita weakened rapidly to a Category 1 intensity with a central pressure of approx. 990 hPa and moved in a southerly direction parallel to the coast at ~ 10 km/h. The system re-entered the Coral Sea north of Townsville early on 13 April and continued moving south-east whilst undergoing extra-tropical transition on 14 April [14, 15]. Meteorological observations, details of Cyclone Ita’s track and sampling and analysis of rainfall and water vapour are summarised in Table 1.

**Table 1:** Sampling, analysis and meteorological observations of Cyclone Ita.

Site	Time of closest approach (AEST)	Distance to eye (km)*	Minimum pressure at site (hPa)	Movement of cyclone (km/hour, direction)	Sampling and analysis
**Lizard Island**	11/04/2014 19:00	0 to +5	954	18, SW	none
**Cape Flattery**	11/04/2014 22:00	0 to +5	963	12, SSW	none
**Cooktown**	12/04/2014 2:00	0 to +15	975	10, S	RF at 2 sites (n = 15)
**Julatten**	12/04/2014 16:00	+5 to +10	No data	10, SE	RF at 2 sites (n = 7)
**Cairns area**	12/04/2014 19:00	+15 to +20	997	10, SSE	Real-time RF and V analysis at 1 site, RF sampling at 8 sites (n = 34)
**Mareeba**	12/04/2014 19:00	-15	998	10, SSE	RF at 1 site (n = 21)
**Goldsborough**	12/04/2014 20:00	-10	No data	10, SSE	RF at 1 site (n = 4)
**Malanda area**	12/04/2014 22:00	-15	No data	15, SSE	RF at 4 sites (n = 17)
**Abergowrie**	13/04/2014 5:00	-30	No data	18, SE	RF at 1 site (n = 4)
**Ingham area**	13/04/2014 6:00	-10 to-20	No data	18, SE	RF at 2 sites (n = 10)
**Townsville area**	13/04/2014 10:00	-15 to-30	997	21, SE	RF at 5 sites (n = 23)

Data from Bureau of Meteorology [14] with the exception of Lizard Island data [16]. RF and V denotes rainfall and vapour sampling, respectively.

*: ‘-’ and ‘+’ indicates land and ocean side of cyclone track, respectively.

**Fig 2:**
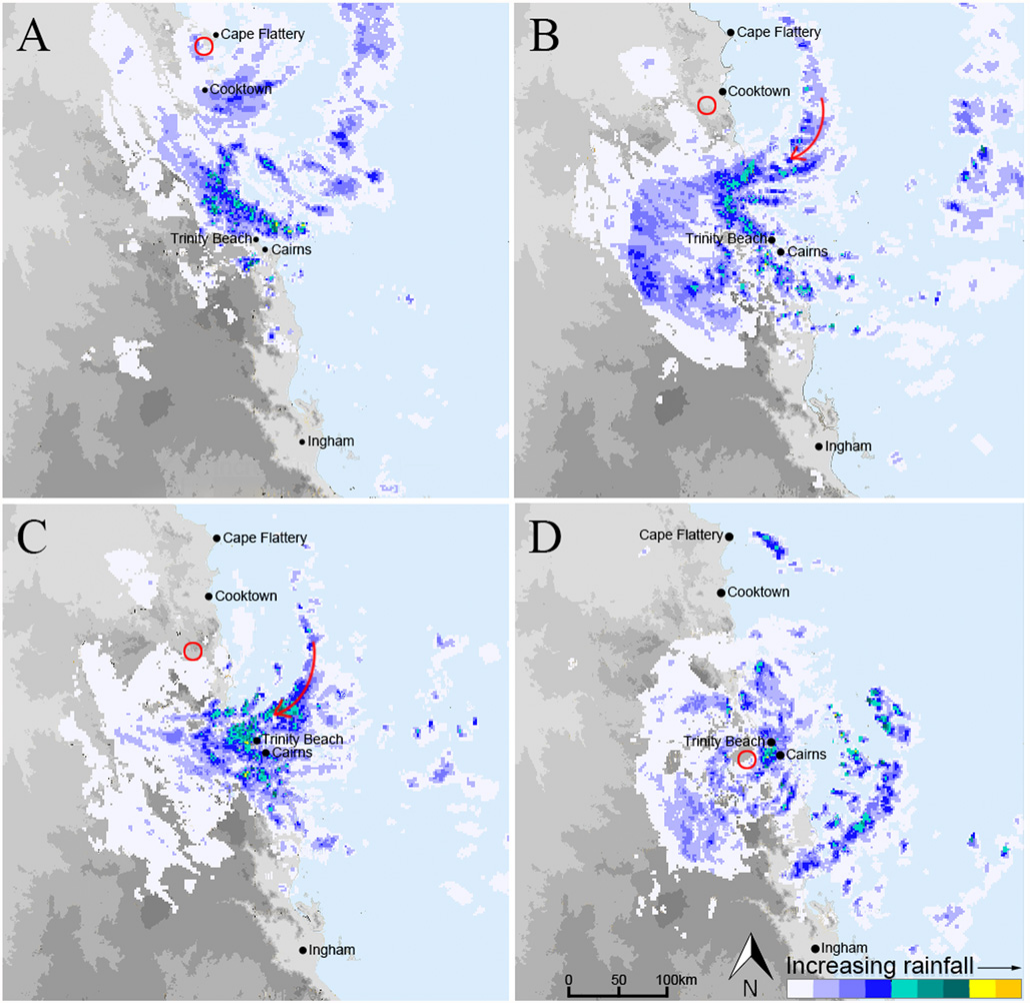
Radar images of TC Ita at 11/4 22:10 AEST (A), 12/4 07:20 AEST (B), 12/4 09:50 AEST (C), 12/4 20:00 AEST (D) [18]. Figure is for representative purposes and is similar but not identical to the original image. The approximate position of the eye of the cyclone is indicated by a red circle. The red arrows indicate a spiral rainband with intense rainfall with the most depleted isotopic composition.

Microwave and radar imaging by NASA’s satellite-borne Tropical Rainfall Measuring Mission (TRMM) show that just prior to landfall on April 11 cloud tops approached an altitude of 15 km near the eye and the most intense rainfall occurred in distinct bands up to an altitude of ~ 6 km [17]. Land-based radar reflectivity images from the WF 100 C Band radar at Cairns [18] show that the cyclone remained relatively well structured with distinct spiral rainbands extending out to a distance of ~200 km over the Coral Sea during its 36 hour transit from Cape Flattery to Townsville (Fig. 2). However, rainbands became poorly defined on the western side of the cyclone as it moved south.

## Methods

### Continuous sampling

Isotopic δ^18^O and δ^2^H values of rainfall were measured continuously at Trinity Beach, Cairns (Lat. 16^o^47.5’ S, Long. 145^o^41.8’ E, altitude 20 m above mean sea level (AMSL)) from April 10–13 2014 using Diffusion Sampling—Cavity Ring-down Spectrometry (DS-CRDS) [19] with addition of thermo-electric control of air and water inlet temperature for enhanced suppression of temperature dependent drift. This system continuously converts rain water into water vapour for real-time stable isotope analysis by a Picarro L2120-i CRDS analyser at 30 s intervals. A total of 1612 30 s measurements of rainfall isotopic composition were acquired during the 28 hour period of rainfall associated with Cyclone Ita. Rainfall was collected on a 0.64 m^2^ inclined metal sheet connected to a small receptacle (~15 ml volume) fitted with a float switch which automatically switched between pumped sampling of rainwater from the receptacle (during rainfall) and reference water (between rainfall) [19, 20]. With a sample uptake of 2.5 ml min^-1^ the collection system provides sufficient rainwater for continuous time-based analysis at a constant rainfall of <1 mm hour^-1^. This design ensures that the receptable volume is rapidly exchanged as rainfall in excess of the pump uptake rate flushes the receptable and flows to waste. However, where rainfall is intermittent the rainfall data may be truncated as 5–10 minutes is required for the isotope measurement to stabilise following a switch from reference water to rainfall.

The raw isotope data was downloaded from the analyser as 30 s average values and corrected for drift by referencing each sample value to two bracketing reference water values. To eliminate memory effects between rapidly changing isotopic compositions, data were omitted from the final results where changes between 30 s values exceeded conservative thresholds limits of 0.2 ‰ for δ^18^O or 1 ‰ for δ^2^H which represent the maximum rate of compositional change that can be captured by the DS-CRDS system. Isotopic compositions are given in the standard δ notation, e.g. δ^18^O = [(^18^O/^16^O_sample_−^18^O/^16^O_standard_) / ^18^O/^16^O_standard_] x 10^3^. Three water standards were analysed three times during the 60 hour observation period through the rainfall uptake system: Lake Eacham Water (δ^18^O = +0.88 ‰; δ^2^H = +3.7 ‰), Evian Water (δ^18^O = -10.64 ‰; δ^2^H = -71.5 ‰) and Casey Snow Melt (δ^18^O = -18.36 ‰; δ^2^H = -140.4 ‰). The isotopic compositions of these standards were determined by WS-CRDS vaporisation analysis (Picarro L2120-i and A0212) and calibrated against the certified IAEA references waters VSMOW, GISP and SLAP. Isotope data precision at a 30 s integration time was typically <0.2 ‰ for δ^18^O and <0.6 ‰ for δ^2^H (1SD). Instrumental drift of the L2120i analyser is expected to be < 0.6 ‰ and < 1.8 ‰ over a 24-hour period for δ^18^O and δ^2^H, respectively [21]. Rainfall intensity was monitored using an Onset HOBO RG3-M logging rain gauge located at James Cook University 2 km from the Trinity Beach site.

Water vapour δ^18^O and δ^2^H values were measured continuously at Trinity Beach using a Picarro L2130-i WS-CRDS. Water vapour isotopic composition was measured at 1 s intervals during the 59 hour approach and passage of Cyclone Ita. Ambient air was introduced to the instrument via a 6 m length of 3.2 mm internal diameter FEP tubing with the inlet located 3 m above ground level under an elevated building and well shielded from ingress of rain. Raw isotope data was downloaded as 30 s average values and scaled to the Vienna—Standard Mean Ocean Water (V-SMOW) using data for water vapour derived from the same three water standards used for scaling the rain fall data. The water standards were quantitatively converted to water vapour using an LGR Water Vapor Isotope Standard Source (WVISS) [22] connected to the Picarro L2130-i analyser before and after the 60 hour observation period. Isotope data precision when analysing a constant vapour source was typically <0.1 ‰ for δ^18^O and <0.2 ‰ for δD (1SD) at a 30 s integration time. Instrumental drift of the L2130i analyser is expected to be < 0.2 ‰ and < 0.8 ‰ over a 24 hour period for δ^18^O and δ^2^H, respectively [21].

### Discrete sampling

Discrete samples (n = 135) of rainfall were collected by volunteers at 27 sites between Cooktown and Townsville (Table 1, Fig. 1, S1 Dataset). At some sites rainfall was collected from roof down pipes (i.e. near-instantaneous grab samples) or from accumulated rainfall in buckets placed on open ground and emptied at ~ 1 hour intervals. At other sites rainfall was accumulated in buckets for ~ 12 hours with scheduled sampling times at 7am and 7pm (AEST). No rainfall samples were obtained from the sparsely populated area west of the narrow coastal strip along TC Ita’s track (Fig. 1).

Samples were analysed using the diffusion sampling WS-CRDS system connected to an auto sampler and scaled to VSMOW as described for the continuous rainfall analysis.

### Synoptic conditions

The Japanese 55-year reanalysis project (JRA-55) dataset [23] were used to examine synoptic scale weather conditions. The JRA-55 data are on a horizontal 1.25 x 1.25 degree grid with 37 vertical layers from 1000 to 1 hPa. By using this data, we calculated the vertically averaged (925–850 hPa) equivalent potential temperature (θ_e_) and vertically integrated (surface to 300 hPa) horizontal water vapor flux (vectors: kg m^-1^ s^-1^).

### Air-mass trajectories

Synoptic scale back-trajectories of air-masses at an altitude of 500 m AMSL were calculated at 6 hourly intervals using the Hybrid Single Particle Lagrangian Integrated Trajectory Model (HYSPLIT) [24] with Global Data Assimilation System (GDAS1) data [25]. Time series of vertical wind and humidity profiles for the Trinity Beach measurement site were also based on data obtained from GDAS1 [25].

### Ethics statement

This study complied with all relevant Charles Darwin University, James Cook University and government regulations. All participants volunteered to participate in the rainfall sampling which took place on their private land. No permissions were required for sampling at these locations. No human, animal (including endangered or protected species) or cell studies were involved and there were no bio-safety implications.

## Results and Discussion

### Rainfall amount and intensity

The total recorded rainfall associated with Cyclone Ita was 198 mm at Cooktown and 211 mm at Townsville [26]. At Trinity Beach we recorded a total rainfall of 231 mm with a rainfall intensity of 7.4 mm hour^-1^ for the continuous rain period from April 11 17:11 to April 13 0:39 (AEST). This intensity slightly exceeds the maximum intensity of 7 mm hour^-1^ recorded for category 1–2 cyclones in a microwave imaging survey of 260 TCs globally [27]. The peak rainfall intensity, recorded during the passage of an inner spiral rainband, was ~13 mm per 10 minutes at Trinity Beach.

### Rainfall isotopes—spatial distribution

The systematic distribution of isotope compositions of rain and vapour within TCs and the direct link between isotope compositions and the physical processes of evaporation and condensation enables O and H isotope compositions to be used as tracers of the dynamics and structural evolution of TCs [6, 10]. Rainfall associated with TCs is usually characterised by δ^18^O and δ^2^H values that are distinctly lower than other tropical rain systems and the isotopic values generally decrease inward towards the core of the cyclone [6, 9, 12]. For example, δ^18^O values in discrete rainfall samples from five TCs in the western Gulf of Mexico ranged from-3.9 to-14.3 ‰ and all samples taken within 100 km of the cyclone eye had δ^18^O values <- 8.7 ‰ [6]. In the Western Pacific, Typhoon Shansan yielded δ^18^O values from ~ -4 to-14 ‰ and δ^2^H values from ~ -20 to-100 ‰ with the lowest values recorded in close proximity to the advancing eye wall of the cyclone [9]. Airborne sampling of TCs has also yielded low isotope ratios in both rain and vapour at altitude [12, 28]. However, in very intense cyclones, the lowest isotope ratios in rain occurred between 50 and 250 km from the eye while isotope ratios were higher in the eye wall due to the incorporation of vapour derived from sea spray [12].

In cyclone Ita the amount-weighted mean isotopic compositions of rainfall at the continuous measurement site at Trinity Beach were δ^18^O = -10.2 ‰, δ^2^H = -67 ‰ (n = 1612) in a total rainfall of 231 mm. The range of isotopic values in rainfall was-4.8 to-20.2 ‰ for δ^18^O and-25.4 to-142 ‰ for δ^2^H whilst the lowest values recorded for a 12 hour cumulative sample were δ^18^O = -19.9 ‰ and δ^2^H = -147 ‰ at Malanda [6]. Due to the high condensation efficiency of the converging surface inflow of moist air masses in TCs the mean isotopic values of TC rainfall can be expected to approach the surface vapour values [6]. This is borne out by the mean δ^18^O and δ^2^H values in TC Ita rainfall which was only slightly higher than the inter-quartile ranges of δ^18^O and δ^2^H values (~ -11 to-13 ‰ and-75 to-90 ‰, respectively) of western Pacific Ocean surface vapour at latitudes of 5 to 25^o^S [29].

While the mean and minimum δ^18^O and δ^2^H values in rainfall during TC Ita are amongst the lowest recorded for a range of different weather systems passing Cairns, a previous convective rainfall event (43 mm total rainfall) associated with the over-land migration of the monsoon trough (Inter-Tropical Convergence Zone) produced mean isotopic values of δ^18^O = -13.8 and δ^2^H = -97 [20]. However, large rainfall amounts (e.g. > 100 mm) with relatively low, but variable, isotopic values are likely to be uniquely associated with TCs [6].

**Fig 3:**
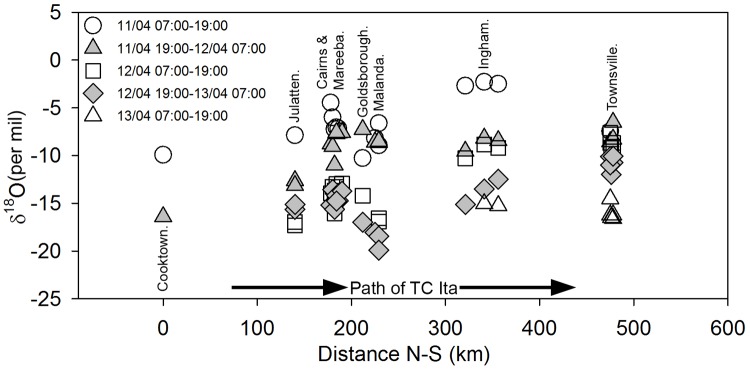
δ^18^O values in discrete 12 hour rainfall samples (n = 85) collected from 27 sites during the north to south passage of TC Ita from April 11–13, 2014.

**Fig 4:**
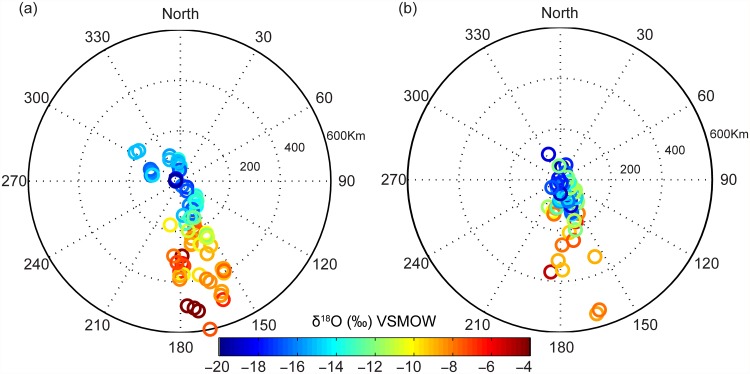
Evolution in space and time of δ^18^O values in discrete (a) 12 hour (n = 85) and (b) 1 hour (n = 50) rainfall samples collected from 27 sites from April 11–13, 2014. δ^18^O values of samples taken at different times are shown as a function of distance and direction from the eye of TC Ita (at centre of plot) at the time of sampling. Some plot positions were moved slightly in order to separate overlapping data.

The distribution of δ^18^O and δ^2^H values in discrete rainfall samples from TC Ita are strongly correlated along the Global and Local Meteoric Water Lines (S1 Dataset); the data will be discussed with reference to δ^18^O values only. The evolution in space and time of δ^18^O values in the 12-hour discrete rainfall samples collected at 27 sites along the path of TC Ita is shown in Fig. 3. The sampling sites covered a distance of approx. 450 km between Cooktown and Townsville and all were within ~ 30 km of the track of the eye of the cyclone (Table 1). It is seen that the range, and progression with time, of isotopic values were similar at all sites as the cyclone approached and passed each site. However, lower minimum δ^18^O values were recorded at the sites at highest altitude (Malanda at ~750 m AMSL ~ -18 to-20 ‰) compared to sites near sea level (Cairns, Ingham and Townsville ~ 16 to-17 ‰. The clear radial distribution of δ^18^O values with δ^18^O < -12 ‰ in all samples collected within 150 km of the eye and, with two exceptions, all δ^18^O values < -8 ‰ within a distance of 400 km from the eye is shown in Fig. 4. Three samples collected more than 500 km to the south of the eye of the cyclone had δ^18^O values > -4 ‰ and represent rainfall prior to the influence of the cyclone. Deuterium excess values (*d* = δ^2^H-8*δ^18^O, S1 Dataset) varied between +6.5 ‰ and +20.8 ‰ (mean = +14.7 ‰) in the discrete rainfall samples but did not vary systematically with distance to the eye of the cyclone.

The data presented in Figs. 3 and 4 demonstrate the similarity in the evolution of the isotopic composition of rainfall at all sampling sites as they were approached and passed by TC Ita and indicate that the structure of the cyclone, its moisture and energy sources remained relatively constant during its 30 hour passage over land. It is likely that the proximity of the track to the coast (< 50 km inland) allowed sufficient inflow of warm and moist oceanic air masses to sustain the energy requirement of the cyclone. This is supported by the cyclone’s intact circular structure as revealed by radar reflectivity maps (Fig. 2a-d) with distinct rainbands on the ocean-ward side as it tracked south whilst the landward side was relatively poorly defined.

### Continuous measurement of rainfall and water vapour isotopes

The high resolution isotope data for rainfall and vapour obtained at Trinity Beach enabled the various influences on the stable isotope evolution of TC Ita to be distinguished.

**Fig 5:**
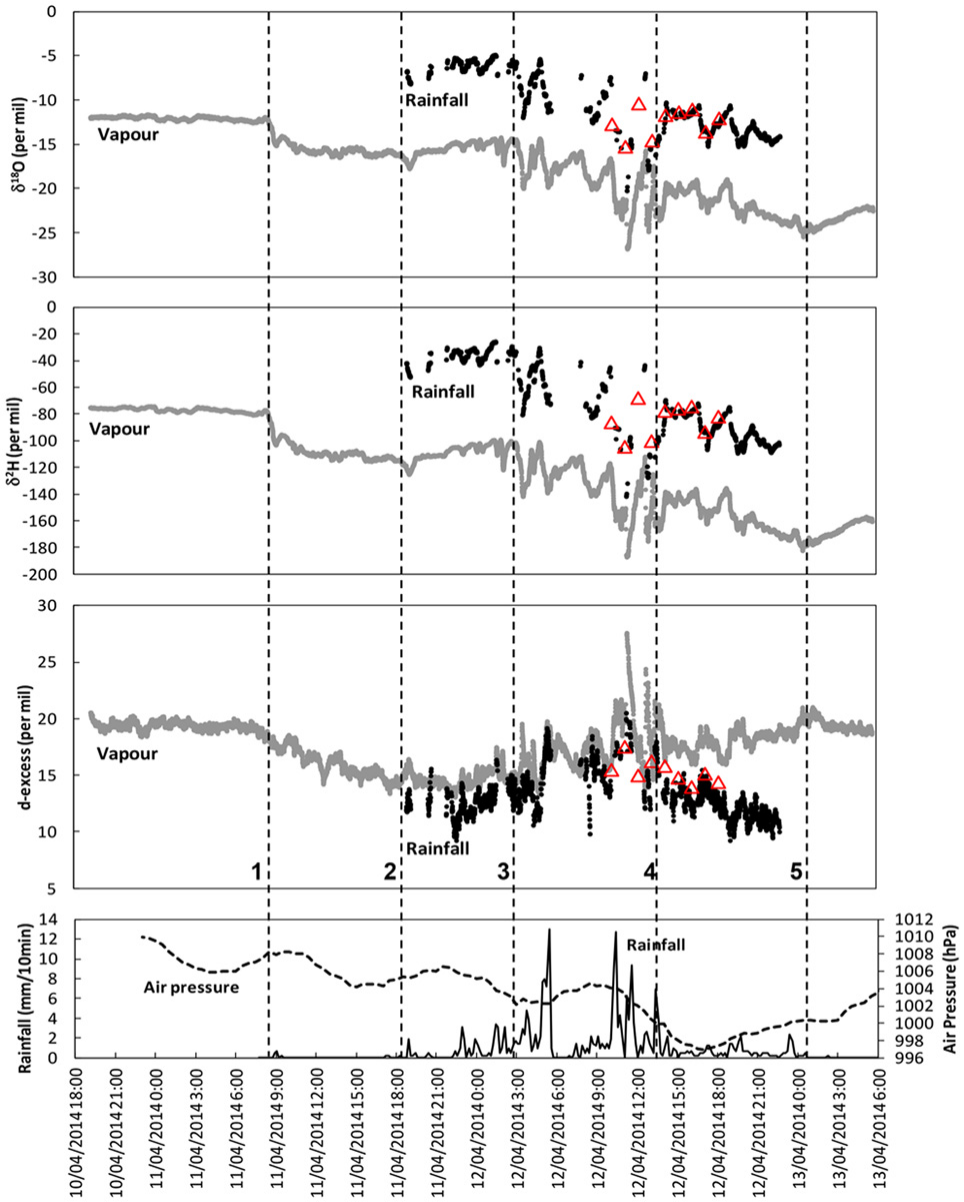
δ^18^O, δ^2^H (30 s interval) and *d*-excess (moving average of five 30 s data points) in water vapour and rainfall and air pressure and rainfall intensity at Trinity Beach. Red triangles indicate values of discrete 1 hour rainfall samples from Trinity Beach. Time markers: 1: First influence of cyclonic circulation; 2: Start of main rain event; 3 to 4: Spiral rain band activity; 5: End of rain event.

Rainfall intensity, δ^18^O, δ^2^H and deuterium excess values are shown in Fig. 5 which also shows the air pressure recorded at nearby Cairns Airport [26]. The data set is provided in S2 Dataset. In addition, the isotope data is interpreted with reference to radar images (Fig. 2) and time series of modelled GDAS-1 wind and moisture profiles at Trinity Beach (S1 Fig.). Furthermore, regional scale air-mass movements were derived using 48 hour HYSPLIT air-mass back-trajectories (S2 Fig.), and JRA-55 regional maps of equivalent potential temperature (θ_e_) and vapour flux (S3 Fig.). It is noted that the 1.25 degrees grid resolution of JRA-55 and GDAS-1 data precludes a reliable analysis of air-mass trajectories close to the cyclone core. Fig. 5 includes isotopic values in 1 hourly accumulated rainfall samples collected at Trinity Beach during part of the measurement period for comparison to the continuous monitoring data.

The most notable features of the continuous vapour and rainfall isotope data associated with TC Ita are (numbered list corresponds to markers ‘1’ to ‘5’ in Fig. 5):

1. A rapid decrease of the vapour d^18^O and δ^2^H values and the commencement of a gradual decrease in vapour *d* value commenced at ~ 9:00 AEST on April 11 coinciding with a short rainfall event (insufficient amount for isotope analysis of rain). The eye of the cyclone was located ~ 370 km to the north over the ocean at this time. HYSPLIT back trajectories show the commencement around this time of an anti-clockwise shift in the source area of air-masses arriving at Trinity Beach. θ_e_ and vapour flux maps show that warm, moist air mass arrived from the northeast around this time.
2. The commencement of the main rainfall event (and the start of rainfall isotope measurement) at ~ 18:00 AEST on April 11. The vapour δ^18^O, δ^2^H and *d* values were relatively constant for several hours around this time. The eye of the cyclone was close to the coastline (Fig. 2a). Air-mass back trajectories show that the air-mass source area continued to move anti-clockwise over the Coral Sea.
3. The commencement at ~ 3:00 AEST on April 12 of the arrival of inner spiral rainbands marked by rapid decreases in δ^18^O and δ^2^H values in both rainfall and vapour and coinciding with intensifications of rainfall (Fig. 2c). *d* values in both vapour and rainfall started to increase around this time. The eye of the cyclone was located ~ 150 km to the northwest over land. Wind speed and relative humidity increased significantly above a height of ~ 950 hPa. The area of highest θ_e_ was centred north of the Trinity Beach measurement site and air-mass back trajectories show air in-flow from this region of highest θ_e_ values.
4. The cessation of rainband activity at ~ 14:00 AEST on April 12. At this time δ^18^O and δ^2^H values rose rapidly in rainfall but not in water vapour and a divergence of *d* values in vapour (continuing to increase) and rainfall (starting to decrease) commenced. The eye of the cyclone was located ~ 60 km to the north-west over land at this time. Within the following 2–3 hours the minimum air pressure (997 hPa) was recorded at Trinity Beach and the wind direction changed towards north-westerly as the eye of the cyclone passed ~ 20 km to the west at ~ 18:00 AEST (Fig. 2d). Moisture levels decreased and arriving air-masses were now tracking over the landmass of Cape York Peninsula.
5. The cessation of rainfall associated with the cyclone at ~ 00:00 AEST on April 13. This was marked by the commencement of rising vapour δ^18^O and δ^2^H values. The eye of the cyclone was located ~ 80 km to the south at this time. Consequently, wind direction gradually changed towards westerly with air masses tracking clockwise over the coastal ranges to the south before descending from the land side towards the Trinity Beach measurement site.

The pronounced parallel tracking of isotopic values in rainfall and vapour in TC Ita during much of the period of rainfall in the advancing front of the cyclone is reflected in strong correlations between δ^18^O and δ^2^H values in rainfall and vapour (R^2^ = 0.97 for both δ^18^O and δ^2^H). This correlation reflects a highly efficient isotopic exchange between rainfall and vapour in a saturated atmosphere. It is notable that vapour isotopic composition prior to the commencement of rainfall remained almost unchanged after rainfall commenced and during the first 8–9 hours of rainfall (Fig. 5 markers ‘1’ to ‘3’). This suggests that the vapour isotopic values measured at ground level until ≈ 3:00 AEST on April 12 were broadly representative of the surface layer inflow. During this period the mean difference in isotopic composition of rainfall (δ^18^O = -6.0 ‰, δ^2^H = -36 ‰) and water vapour (δ^18^O = -15.3 ‰, δ^2^H = -108 ‰), i.e. δ^18^O_rain-vapour_ = 9.3 ‰, δ^2^H_rain-vapour_ = 72 ‰, corresponded closely to the equilibrium liquid-vapour fractionation (10^3^lnα_l-v_(^18^O) = 9.2–9.4, 10^3^lnα_l-v_(^2^H) = 74–77 [30] within the range of surface temperature observed at Trinity Beach during TC Ita (24.5–26.5°C).

The rapid decrease of vapour δ^18^O and δ^2^H values ~ 9:00 AEST on April 11 (Fig. 5, marker ‘1’) coincided with the arrival of a tropical air mass with high θ_e_ value. The decrease of δ^18^O and δ^2^H values in this tropical air mass is consistent with previous isotope data collected along 20 oceanic transects over a 4-year period which showed that isotopic values of surface vapour in the tropics is significantly lower than those in the subtropics [29].

The rapid decrease in δ^18^O and δ^2^H values of both rainfall and vapour from ~ 3:00 to 14:00 AEST on April 12 (Fig. 5, markers ‘3’-‘4’) were associated with the arrival of an inner spiral rainband. This rainband was sustained for several hours and included several convection cells with anvil stratiform rainfall regions expanded towards the outside of the core by the tangential winds (see radar reflectivity maps in Fig. 2b and c). It has previously been shown that stratiform rain in hurricanes (cyclones) has significantly lower isotope ratios than convective rain as a result of its higher mean altitude of condensation and larger percentage of precipitation derived from great heights [12]. In the inner spiral rainbands of cyclones there is a successive cycling of moisture through subsidence under stratiform rainfall and transport of isotopically depleted vapour into the lower troposphere and towards the TC center where it is reused in subsequent convective condensation-precipitation cycles (cf. [29, 31, 32]). Consequently, the isotopic composition of precipitation becomes gradually depleted towards the TC center as the contribution of recycled water increases along the rainband. As the eye of the cyclone approached the Trinity Beach measurement site, the cycling of moisture through successive rainfall events along the upstream region of the spiral band ceased and thus the δ^18^O and δ^2^H values in rainfall rapidly increased (just prior to marker ‘4’ in Fig. 5). In contrast, this rapid increase was not seen in vapour.

The general trends of δ^18^O and δ^2^H values in rainfall and vapour continued to decrease after the main rainband passed Trinity Beach ~ 14:00 AEST on April 12 (Fig. 5, markers ‘4’ to ‘5’) and after the passage of the core of TC Ita to the west (marked by the minimum recorded air pressure, Fig. 5). As the TC moved further south the air mass reaching Trinity Beach after 14:00 AEST on April 12 passed over the coastal ranges to the south in a clockwise rotational motion over the landmass of Cape York Peninsula. This low-level flow passed through the upstream region of the spiral rain band (positioned to the south of Trinity Beach at this time, Fig. 2d) and may have transported moisture with low δ values and high *d* value derived from the air mass subsidence under stratiform rainfall regions. An additional contributing factor to the observed lowering of δ values may have been the addition of land-derived moisture which was derived from earlier low-δ precipitation in the advancing front of TC Ita itself.

### Deuterium-excess

The *d* value in water vapour is often used as a tracer of moisture origin because the *d* value of moisture evaporated from the oceanic surface depends on surface humidity, temperature and wind speed [13, 33, 34] with humidity the most important factor. The *d* value decreases with increased relative humidity over the ocean and with decreasing temperature of the ocean surface. In the tropics, increases in surface vapour *d* values may also indicate subsidence of air from the upper troposphere [29, 32]. The production of precipitation from water vapour essentially conserves the *d* value [35] although falling raindrops may partially evaporate or re-equilibrate isotopically with surrounding vapour during descent [36, 37].

The decrease in *d* values (Fig. 5 markers ‘1’ to ‘2’) in vapour at Trinity Beach as the outer circulation envelope of TC Ita approached can be attributed to the accelerating inflow of moisture derived from evaporation of surface waters at high relative humidity and temperature as indicated by high θ_e_ values (S3 Fig.). However, as TC Ita approached the measurement site rainfall intensified and *d* values in both vapour and rainfall increased (Fig. 5, markers ‘3’ to ‘4’). Previous simulations of the isotopic evolution of hurricanes (cyclones) have shown that *d* values in rainfall increase near the eye although the simulated *d* values were lower than observed during TC Ita [12]. In addition, similar increases in *d* values, accompanied by decreasing δ^18^O and δ^2^H values has been observed during the active convective phase of Madden-Julian Oscillations (MJO) in the tropical atmosphere [32]. They highlight the role of vapour recycling due to the subsidence of air masses from stratiform clouds. Because the lowest δ^18^O and δ^2^H values during TC Ita corresponded to the successive linked convective-stratiform rainfall events (Figs. 2b-c and 5), the large increase of *d* values may be attributed to downward moisture transport above the boundary layer.

A marked divergence in the *d* values of vapour and rain occurred from ~ 14:00 AEST on April 12 (Fig. 5 marker ‘4’) and continued after the passage of the eye of TC Ita. The divergence in *d* values indicates that moisture sources were different for precipitation and surface vapour. As described above air masses that arrived after 14:00 AEST ascended the coastal mountain range south of the measurement location, travelled in a clockwise direction across the hinterland and descended the ranges towards the Trinity Beach measurement site. We surmise that the low-level air flow through the upstream region of the spiral rain band (positioned to the south of Trinity Beach at this time, Fig. 2d) supplied surface moisture with low δ values and high *d* values whereas precipitation from higher levels was becoming less depleted and had relatively low *d* values as it was no longer derived from successive rainband activity.

Enhanced sub-cloud evaporation may also have played a limited role in decreasing *d* values of falling rain as the measurement site at Trinity Beach is located on a narrow coastal strip adjacent to an elevated hinterland. Observed dewpoints at Mareeba on the hinterland were nearly 5°C lower than at the coast during this period [26]. It appears that orographic rain south of Trinity Beach dried out the air mass as it moved clockwise over the elevated ranges in that region. Once this relatively dry air mass descended the range it dried further, thereby enhancing sub-cloud evaporation and decreasing the *d* values of falling rain.

### Relevance to speleothem isotope records

There has been uncertainty about the relative importance of various parameters in a tropical cyclone such as intensity, longevity and distance to the eye in terms of the isotope signal recorded in palaeo-archives. Previous research [1] compared these parameters to annual isotope signals recorded in a stalagmite over the past ~100 years and showed that the strongest relationship was between TC intensity divided by the distance of the cyclone to the sample site and the isotope signal. The isotope values measured during TC Ita also suggest that distance was an important factor. Although the central pressure of TC Ita increased considerably shortly after crossing the coast, the spatial pattern of rainfall δ^18^O and δ^2^H values surrounding the cyclone as it approached and passed a measurement site remained remarkably constant along a 450 km long path over land (Fig. 4). It remains uncertain whether this relationship was a function of distance alone or in combination with decreasing air pressure as TC Ita approached and passed each site. However, it is clear that distance (and likely intensity) is important in determining the isotope values in TC rainfall and the isotopic signal imparted in speleothem limestone deposits. Similar studies of future TCs of varying intensity, longevity and coastal crossing locations should help refine the tempestological interpretation of stable isotope signatures found in speleothems and other palaeo-archives.

## Conclusions

Continuous measurement in real time of δ^18^O and δ^2^H values in both rainfall and water at a single site coupled with measurement of discrete rainfall samples from multiple sites provided a detailed characterisation of the stable isotope anatomy of TC Ita. In conjunction with local and synoptic meteorological observations the stable isotope values could be linked to specific features of the cyclone such as the passage of convective spiral rainbands, stratiform rainfall and the arrival of a succession of subtropical and tropical air masses with changing oceanic and continental moisture sources.

This study demonstrates that the stable isotope anatomy of TCs can be linked to the detailed physical evolution of the cyclone as well as to their synoptic-scale meteorological setting. At the continuous measurement site the near-simultaneous variations in δ^18^O and δ^2^H values in rainfall and water vapour and an approach to liquid-vapour isotope fractionation equilibrium indicated isotopic exchange between rainfall and vapour during the approach of TC Ita. Following the passage of spiral rainbands and the cyclone eye, different moisture sources for rainfall and vapour were reflected in diverging d-excess values.

The delineation of the magnitude, spatial scale and longevity of the isotope anomaly associated with TC Ita confirms previous assertions that intense, isotopically depleted rainfall from TC’s is likely to impart a detectable isotope signal in a range of environmental proxies over a significant area.

Stable isotope data acquired at high temporal resolution will also provide detailed insights into the hydrological cycle of TCs.

## Supporting Information

S1 Dataset Rainfall isotope data for discrete samples including information on sampling locations. (ODS)

S2 Dataset Continuous rainfall and vapour isotope data and rainfall intensity at Trinity Beach. (ODS)

S1 Fig Wind and humidity profiles at Trinity Beach during the approach and passage of TC Ita April 10–13 2014. Top: Relative humidity (%) is shown by colour scale. Bottom: Wind speed is shown by colour scale and direction by compass arrows. Based on data obtained from GDAS1 [25].
(TIF)

S2 Fig HYSPLIT 48-hour back-trajectories [24] of air masses reaching Trinity Beach at an altitude of 500 m AMSL during TC Ita. Trajectories with tick marks at 6 hourly intervals are labelled with arrival date and time (AEST).
(TIF)

S3 Fig Vertically averaged (925–850 hPa) equivalent potential temperature (θ_e_) contour maps and vertically integrated (surface to 300 hPa) water vapour flux (white vectors: kg m^-1^ s^-1^) at 12 hourly intervals from April 10, 2014 16:00 (AEST) to April 13, 2014 04:00 (AEST) based on the JRA-55 dataset [23]. The Trinity Beach measurement site is indicated by a white circle.
(TIF)
