# Supplementary figures and images for: Stable Isotope Anatomy of Tropical Cyclone Ita, North-Eastern Australia, April 2014
Source: PLoS One. 2015 Mar 5;10(3):e0119728. doi: 10.1371/journal.pone.0119728 (PMC4351091; doi:10.1371/journal.pone.0119728)

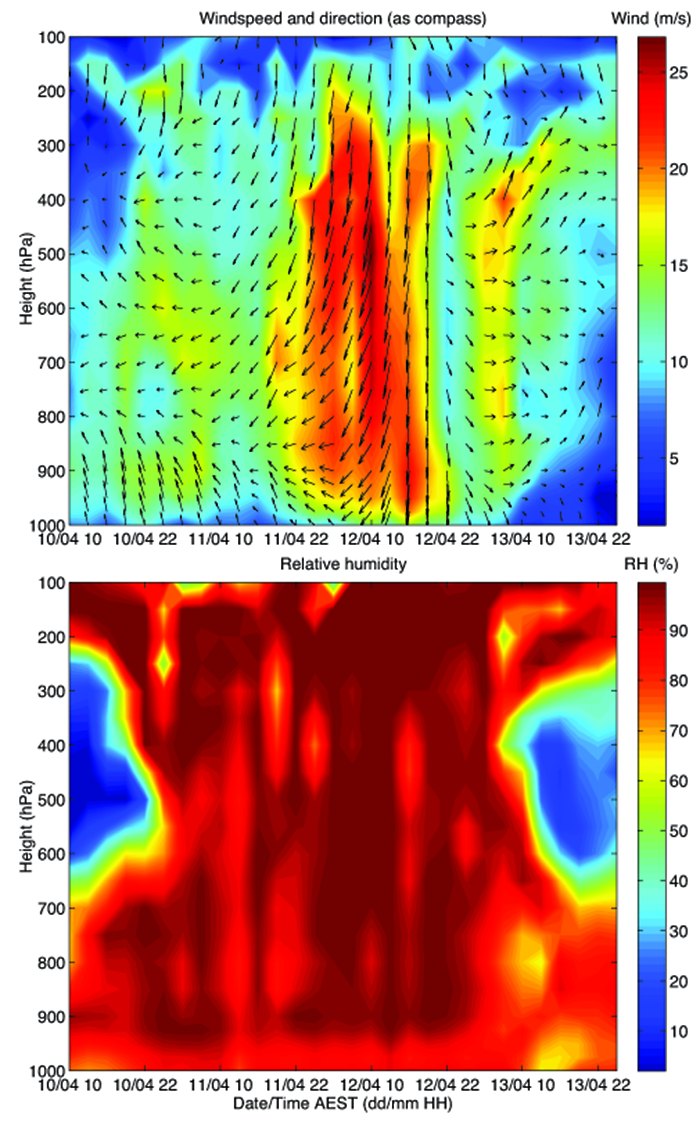

Supplement: S1 Fig — Top: Relative humidity (%) is shown by colour scale. Bottom: Wind speed is shown by colour scale and direction by compass arrows. Based on data obtained from GDAS1 [25]. (TIF) [file pone.0119728.s003.tif]

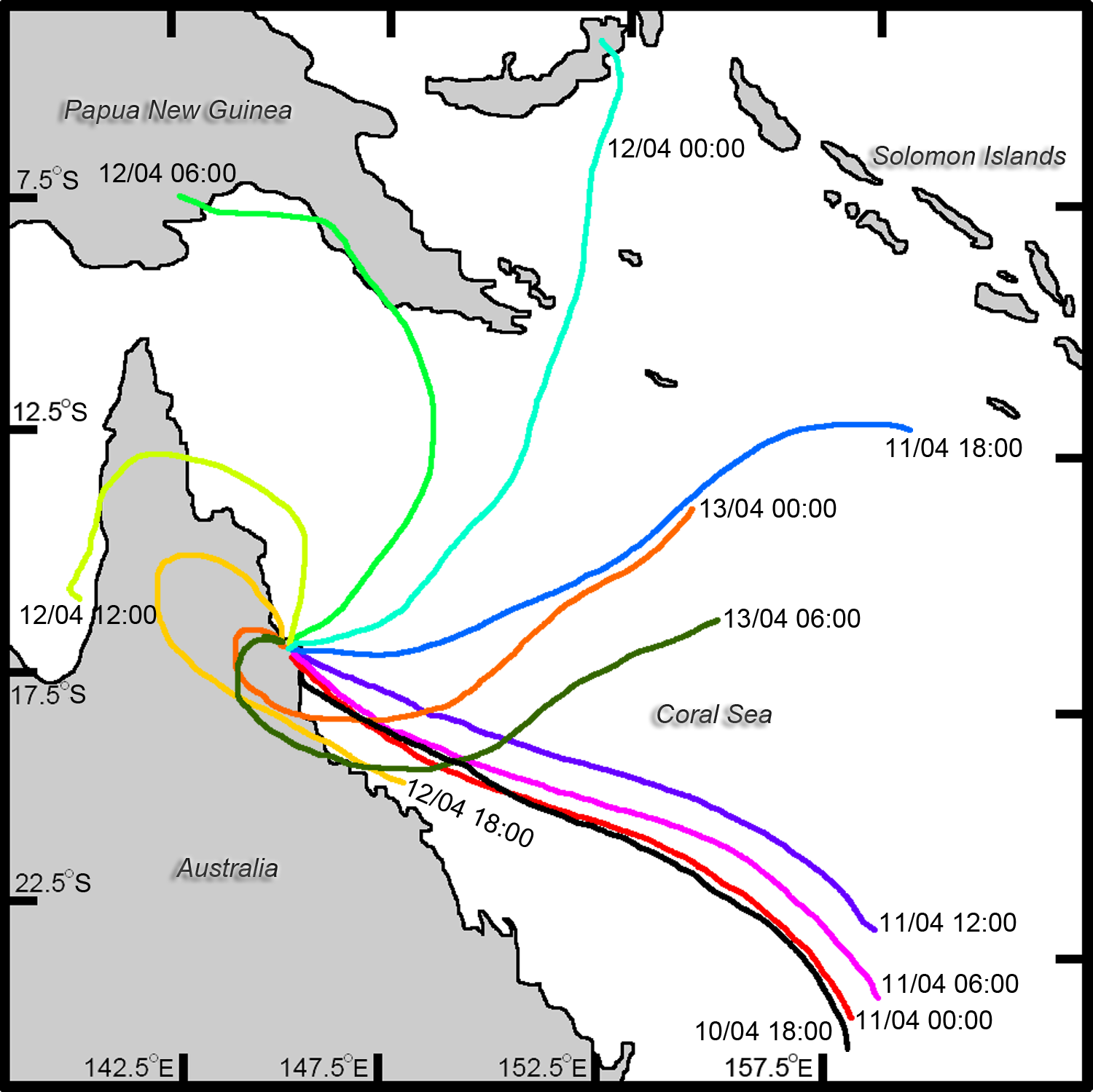

Supplement: S2 Fig — Trajectories with tick marks at 6 hourly intervals are labelled with arrival date and time (AEST). (TIF) [file pone.0119728.s004.tif]

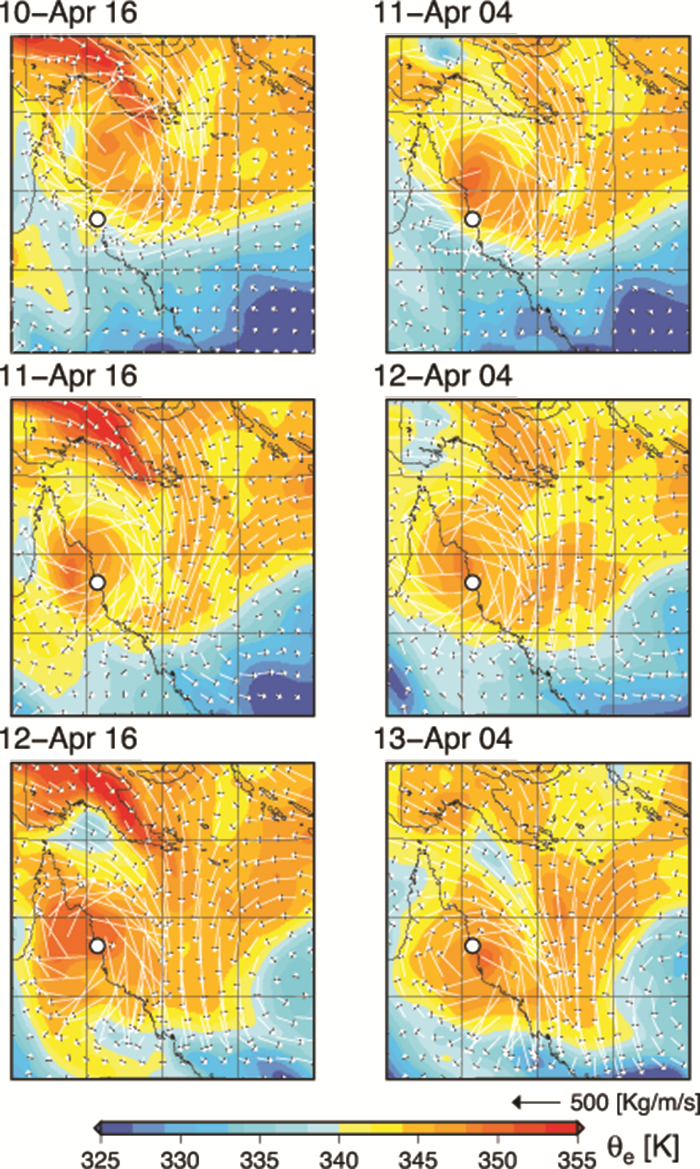

Supplement: S3 Fig — The Trinity Beach measurement site is indicated by a white circle. (TIF) [file pone.0119728.s005.tif]
